# Supplementary material for: Resolving the full spectrum of human genome variation using Linked-Reads
Source: Genome Res. 2019 Apr;29(4):635–45. doi: 10.1101/gr.234443.118 (PMC6442396; doi:10.1101/gr.234443.118)
Supplement: Supplemental Material [file supp_gr.234443.118_Supplemental_longranger-2.2.zip › longranger-2.2.tar.bz2!/lr2.2-public/lib/stan/cmdstan/stan/doc/api/html/common__adaptors__def_8hpp_source.html]

Stan: src/stan/gm/grammars/common\_adaptors\_def.hpp Source File


|  |  |
| --- | --- |
|  | Stan  2.5.0  probability, sampling & optimization |


- Main Page
- Related Pages
- Namespaces
- Classes
- Files

- File List
- File Members

All Classes Namespaces Files Functions Variables Typedefs Enumerations Enumerator Friends Macros Pages

- src
- stan
- gm
- grammars

common\_adaptors\_def.hpp

Go to the documentation of this file.

1 #ifndef STAN\_\_GM\_\_PARSER\_\_COMMON\_ADAPTORS\_DEF\_\_HPP

2 #define STAN\_\_GM\_\_PARSER\_\_COMMON\_ADAPTORS\_DEF\_\_HPP

3

4 #include <boost/fusion/include/adapt\_struct.hpp>

5

6 #include <stan/gm/ast.hpp>

7

8 BOOST\_FUSION\_ADAPT\_STRUCT(stan::gm::range,

9  (stan::gm::expression, low\_)

10  (stan::gm::expression, high\_) )

11

12

13 #endif

stan::gm::range

**Definition:** ast.hpp:401

stan::gm::expression

**Definition:** ast.hpp:216

BOOST\_FUSION\_ADAPT\_STRUCT

BOOST\_FUSION\_ADAPT\_STRUCT(stan::gm::expr\_type,(stan::gm::base\_expr\_type, base\_type\_)(size\_t, num\_dims\_))

ast.hpp


---

[ Stan Home Page ]

*© 2011–2014,
Stan Development Team.*
